# Click-to-Capture: A method for enriching viable *Staphylococcus aureus* using bio-orthogonal labeling of surface proteins

**DOI:** 10.1371/journal.pone.0234542

**Published:** 2020-06-17

**Authors:** Aryaman Shalizi, Toni N. Wiegers, Hédia Maamar

**Affiliations:** Department of Assay Development, Talis Biomedical Corporation, California, United States of America; The University of Jordan School of Pharmacy, JORDAN

## Abstract

*Staphylococcus aureus* is one of the principal causative agents of bacteremia which can progress to sepsis. Rapid diagnostic tests for identification and antibiotic resistance profiling of *S*. *aureus* would improve patient outcomes and antibiotic stewardship, but existing methods require a lengthy culture step to obtain enough material for testing. Complexity of the host matrix, where pathogenic microbes are often present, also interferes with many diagnostic methods. Here, we describe a straightforward and rapid method for enriching viable *S*. *aureus* using bio-orthogonal, or “click,” chemistry methods. Bacteria labeled in this manner can potentially be cultured, interrogated using molecular methods for pathogen identification, or used to test antibiotic susceptibility.

## Introduction

Sepsis is a life-threatening organ dysfunction caused by a dysregulated response to infection and represents a growing problem for clinical medicine [1]. Globally, it is estimated that sepsis affects 30 million people annually, and roughly 1 in 4 individuals who become septic die [2]. Because of its swift and severe progression, critical care guidelines recommend initiation of antimicrobial therapy within one hour of diagnosis [3]. This empiric treatment is initiated with one or more broad-spectrum antimicrobials and may include antifungal and antiviral agents as well. However, such life-saving interventions are not without risks: disruption of the normal microbiota by broad-spectrum antibiotics has been linked to a variety of chronic health conditions, as well as infections by opportunistic pathogens such as *Clostridium difficile* [4, 5].

In addition to the human and economic toll exacted by sepsis, ineffective sepsis treatment contributes to the emergence of antibiotic resistant microbial strains. Consequently, it is expected that mortality due to antibiotic resistance will become one of the leading causes of death globally by mid-century [6, 7]. Over the last three decades, gram positive bacteria have emerged as a leading cause of sepsis, in both hospital and community acquired settings [8, 9]. The opportunistic pathogen *Staphylococcus aureus* is the most frequently identified gram-positive organism, and one of the top three organisms overall, in culture-positive cases of sepsis [10]. Of particular concern is the global spread of antibiotic resistant strains of *S*. *aureus* [9–12]. Although there are substantial regional variations in prevalence of methicillin resistant *S*. *aureus* (MRSA) and vancomycin intermediate or resistant *S*. *aureus* (VISA and VRSA, respectively), resistant strains account for more than 50% of clinical isolates in some settings [12, 13].

The spread of resistant strains has dire health and economic consequences, including longer and more expensive hospital stays, increased likelihood of mortality during hospital stays and following discharge, and increased incidence of chronic health conditions among survivors [14, 15]. Rapid diagnostic tests capable of accurately identifying specific pathogenic agents and their antibiotic sensitivities are essential for reducing clinical reliance on broad-spectrum antimicrobials. As such, the development of rapid diagnostic methods is central to any long-term antibiotic stewardship strategy [9].

The rapid identification of pathogenic organisms from biological samples is a central problem of laboratory diagnostics. Because antibiotic resistance in *S*. *aureus* is typically associated with the acquisition of a single resistance marker through horizontal gene transfer–*mecA* for methicillin resistance, and *vanA* for vancomycin resistance–direct genotypic assays could in principle be sufficient for identifying the resistance profile of a given clinical isolate [16]. The development and implementation of direct-from-blood assays for antibiotic resistance faces numerous obstacles: Pathogens are present in a complex milieu of host cells and macromolecules, many of which interfere with methods for growth and detection of microorganisms; furthermore, pathogens may be present at very low abundance in patients with clinically-defined sepsis–less than 1 CFU/mL according to some reports [17]. As a consequence, the standard-of-care for laboratory diagnostics is laboratory culture of the causative organism from large-volume blood draws, followed by serial microdilution for antibiotic sensitivity testing. Even “rapid” molecular assays currently on the market begin from culture-positive samples containing upwards of 10^6^ CFU/mL of the suspected pathogen [18].

A rapid method for isolating *S*. *aureus* from patient samples would represent a substantial improvement for sepsis care. Concentrating low-abundance bacteria from a large volume may allow for the collection of sufficient material for molecular identification without the added wait time for a culture-positive sample. Furthermore, enriching viable bacteria from a large sample volume may allow for the implementation of rapid-turnaround antibiotic susceptibility testing on small cell populations.

One possible avenue for microbe isolation is affinity-based enrichment using specific antibodies or pattern recognition proteins (PRP) of the innate immune system, such as C-type lectins [19]. These methods rely on the recognition of pathogens by specific surface properties, but may be limited by the inherent specificity of the antibody or PRP used for enrichment; no antibody or PRP can recognize all target organisms [20]. Within *S*. *aureus*, genetic heterogeneity in cell wall composition contributes to immune evasion and directly affects interaction with components of both innate and adaptive immune systems [21, 22].

A promising approach to concentrate *S*. *aureus* from blood, described in this manuscript, is the use of bio-orthogonal, or “click,” chemistry methods [23]. These methods rely upon the incorporation into nascent biopolymers of nucleotides [24], amino-sugars [25], or amino-acids [26], derivatized with a reactive azide or alkyne group. In strain-promoted azide-alkyne cyloaddition (SPAAC), biopolymers containing azide-substituted moieties react spontaneously in physiological solutions with cyclooctyne compounds that incorporate additional functionalities of interest, such as fluorophores or affinity probes [27, and references therein].

The potential of click-based methods for enrichment of intact microorganisms has been shown using Kdo-Azide, an azido-variant of keto-deoxyoctulosonate (Kdo), one of the core components of bacterial lipopolysaccharide (LPS) [28]. One limitation of this method is that labeling with Kdo-Azide is comparatively slow, with several hours of labeling required for effective enrichment. Furthermore, it is only effective in gram-negative organisms that utilize LPS in their outer membranes.

In this study we examined the feasibility of a metabolic labeling approach for enrichment of the gram-positive organisms *S*. *aureus*. We identified bio-orthogonal small molecules that are rapidly incorporated by *S*. *aureus* and identified critical parameters of linker structure and magnetic bead geometry for the effective capture of viable organisms. We also demonstrated the capacity of this method for labeling in, and enrichment from, a contrived blood sample.

## Materials and methods

### Strains and reagents

The *S*. *aureus* strain ATCC 25923 was used for all experiments. The metabolic labeling agents Azido-homoalanine (LAHA), N-azidoacetylglucosamine tetracetylated (GNAZ), N-azidoacetylmannosamine tetracetylated (MNAZ), N-azidoacetylgalactosamine tetracetylated (GLAZ), DBCO-MB-543 octyne-fluorophore (DM543), and water-soluble DBCO-sulfo-biotin (DSB), were obtained from Click Chemistry Tools (Scottsdale, AZ). 3-Azido-D-alanine HCl (DALA) was obtained from Jena Bioscience (Jena, Germany). ProLong Diamond hard-set mounting medium, DBCO-PEG_n_-Biotins (30-, 60- and 100-PEG linkers), M280 and MyOne T1 streptavidin beads, Ca- and Mg-free Dulbecco’s Phosphate Buffered Saline (DPBS), Fetal Bovine Serum (FBS), Pronase, and Streptavidin-AlexaFluor 546 (SAF546) were obtained from Fisher Scientific (Waltham, MA). Super Mag 0.2 μm, Hi-Sur Mag 1 μm and Hi-Sur Mag 0.15 μm streptavidin beads were obtained from Ocean NanoTech (San Diego, CA). DNase I was obtained from Worthington Biochemical (Lakewood, NJ). M9 minimal medium, Saponin, Tween-20, 37% v/v paraformaldehyde, and 0.1% w/v poly-L-lysine (PLL) were obtained from Sigma-Aldrich (St. Louis, MO). Tryptic Soy Broth (TSB) and Tryptic Soy Agar (TSA) plates were obtained from Teknova (Hollister, CA)

### Metabolic labeling for direct fluorescence

All growth steps were performed at 37°C unless otherwise indicated. Overnight cultures were seeded in TSB from a single bacterial colony grown on solid medium (TSA). The overnight culture was diluted 1:100 in M9 minimal medium and grown to mid-log phase (OD 0.5–0.7 at 600 nm). Mid-log cultures were grown with 5 mM of the indicated azido-metabolic label or DMSO vehicle for 1 hour, unless otherwise specified in the text or figure legends. The cells were pelleted by centrifugation (12,000 x *g*/2 minutes), and the pellet was washed twice with wash buffer (WB; 2% v/v FBS, 0.05% v/v Tween-20, and DPBS). Fluorophore addition was done with 20 μM of DM543 or DMSO control in WB for 30 minutes at room temperature with continuous agitation. After fluorophore addition, cells were pelleted by centrifugation, washed twice with WB, and resuspended in 4% v/v paraformaldehyde to fix. After fixation, cells were pelleted by centrifugation, washed twice with DPBS, plated to PLL-coated coverslips, and immobilized with hard-set mounting medium.

### Metabolic labeling for indirect fluorescence or enrichment

For experiments done in growth medium, cells were metabolically labeled as described above. After washout of the azido-metabolic label, cells were incubated in WB for 30 minutes at room temperature with continuous agitation in the presence 20 μM of DBCO-biotin or DMSO vehicle. The specific linker types for each DBCO-biotin are indicated in the figure legends and text.

For experiments done in blood, 0.5 mL of mid-log culture was spiked directly into 5 mL of heparinized blood. Host cells were lysed by the addition of a proprietary detergent-enzyme cocktail. Detergent-enzyme treatment was done simultaneously with the addition of 5 mM LAHA for metabolic labeling, which was done for 1 hour at 37°C with continuous agitation, unless otherwise specified in the figure legends or text. Cells were collected by centrifugation, the supernatant discarded, and the pellet washed twice with WB. The pellet was resuspended in WB and incubated with DBCO-biotin or DBCO-fluorophore as described above.

For microscopy, after biotin addition, cells were pelleted by centrifugation, washed twice with WB, and incubated for 1 hour at room temperature with continuous agitation in the presence of 20 μg/mL SAF546. Cells were pelleted by centrifugation to remove unbound streptavidin, the pellet was washed twice with WB, and resuspended in 4% v/v paraformaldehyde to fix. After fixation, cells were mounted for microscopy as described above.

For enrichment, after biotin addition, cells were pelleted by centrifugation, washed twice with WB, and resuspended in 1 mL WB. An aliquot was retained to determine input CFU by plating on solid medium. The remaining sample was incubated 1 hour at room temperature with continuous agitation with 100μg of streptavidin magnetic beads in 1 mL of WB. The particular streptavidin bead used is specified in the text and figure legends. Beads were collected on a neodymium magnet, and the unbound supernatant was retained to assay depletion efficiency by plating on solid medium. Beads were washed twice and resuspended in WB, then plated on solid medium to assay enrichment efficiency.

### Microscopy and image analysis

Image acquisition was performed on a Nikon Eclipse Ti microscope with automated stage controlled by NIS Elements software, using a 100x oil-immersion objective. Images were captured as 7-slice Z-stacks with an offset of 0.25μm between slices. Images were analyzed using the open-source FIJI package. Z-slices were summed into a single image, and a mask based on the brightfield image was used to analyze the fluorescence intensity of individual cells. A circularity range of 0.6–1.0 was used. Population fluorescence histograms were generated in Excel.

### Colony formation assay

Samples from input, bound, or supernatant fractions were plated to solid medium (TSA) using roller beads. Two plates were prepared for each condition. After shaking, the beads were discarded, and plates were left overnight at 37°C with atmospheric CO_2_. The following morning, total colony number was determined using a Flash & Go colony counting system. Enrichment efficiency was determined by dividing the observed CFU on plates from the bound fraction over the observed CFU on plates from the input fraction. Depletion efficiency was determined by dividing the observed CFU on plates from supernatant fraction over the observed CFU on plates from the input fraction

## Results and discussion

**Fig 1:**
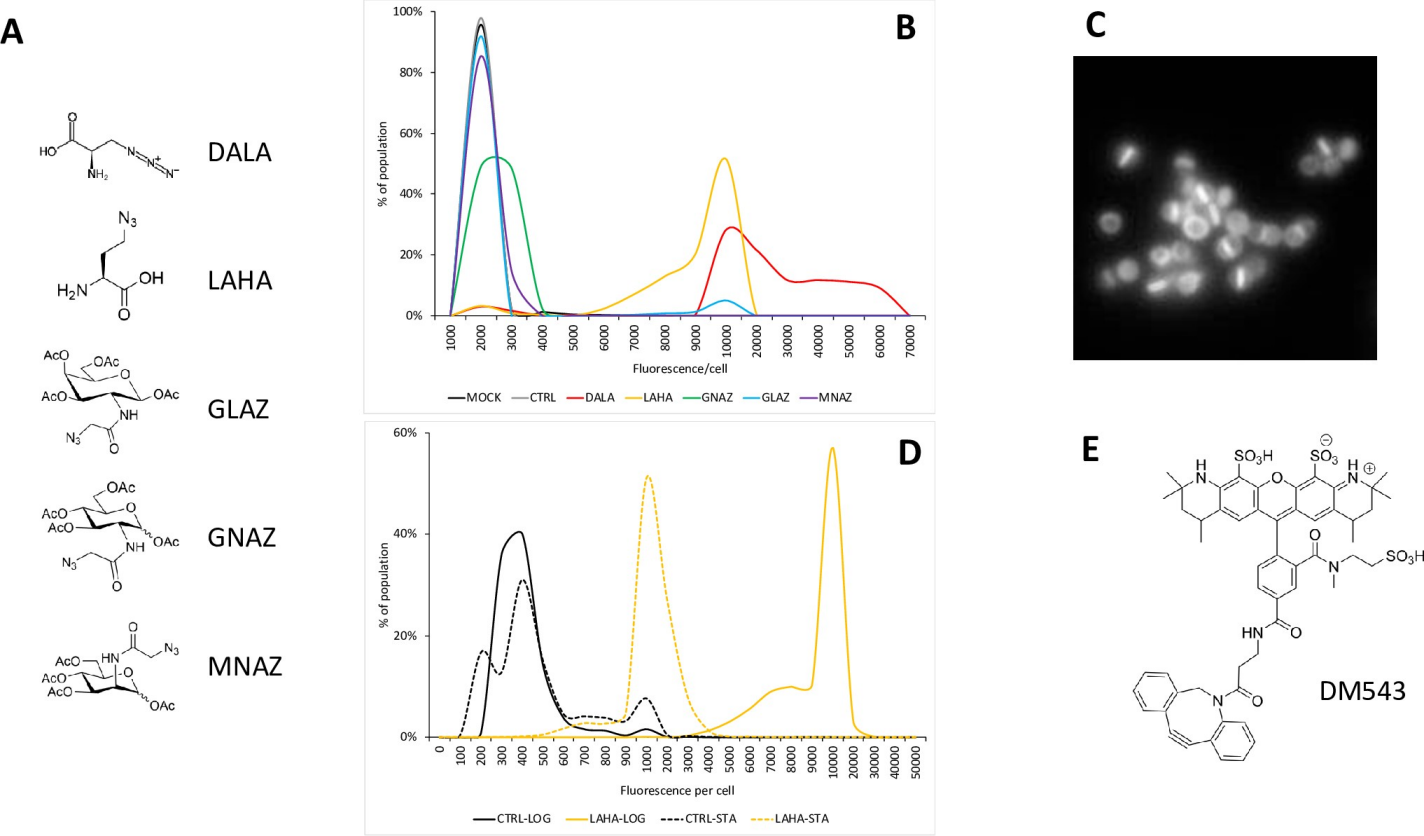
LAHA can specifically label *Staphlyococcus aureus*. (A) Molecular structures of the metabolic labels used in this study; DALA = Azido-D-Alanine; LAHA = L-Azidohomoalanine; GLAZ = Azidogalactosamine; GNAZ = Azidoglucosamine; MNAZ = Azidomannosamine. (B) LAHA robustly labels *S*. *aureus*, as detected by direct cycloaddition of the fluorophore DBCO-MB-543 (DM543). MOCK = cells grown without exposure to metabolic label or DM543; CTRL = cells grown without metabolic label, but exposed to DM543 15 minutes; DALA, LAHA, GNAZ, GLAZ, MNAZ = cells exposed to 5 mM of the indicated metabolic label for 1 hour, followed by 15 minutes exposure to DM543. (C) Distribution of incorporated metabolic label, primarily at the equatorial plane of *S*. *aureus*. (D) LAHA labels *S*. *aureus* during log-phase (LOG, solid line) as well as stationary phase (STA, broken lines); CTRL = cells grown without metabolic label, but exposed to DM543 15 minutes (black lines); LAHA = cells grown with LAHA for 1 hour followed by 15 minute exposure to DM543 (yellow lines). n = 3 for each experiment; a representative example is shown for each. (E) Molecular structure of fluorophore dibenzocyclooctyne DBCO-MB-543 (DM543).

To develop a method for bacterial enrichment using SPAAC, we screened a panel of azido-modified metabolic building blocks for rapid incorporation in *S*. *aureus* strain ATCC 25923. The panel included the amino sugars azido-mannosamine (MNAZ), azido-glucosamine (GNAZ), and azido-galactosamine (GLAZ), which can participate in a variety of cell-wall synthesis and protein glycosylation pathways; the D-amino acid azido-D-alanine (DALA), which is incorporated into the cell wall; and L-azidohomoalanine (LAHA), which substitutes for methionine in newly-synthesized proteins (Fig 1A). Log-phase cells were incubated for 1 hour in minimal medium supplemented with 5 mM of an azido molecule or DMSO vehicle control. After washout of the unincorporated metabolic label, cells were stained with a fluorophore dibenzocyclooctyne (DM543, Fig 1E). Unincorporated fluorophore was washed away, the cells were fixed with formaldehyde, and examined by fluorescence microscopy.

Compared to control cells grown without metabolic label, we found that both amino acid derived labels, LAHA and DALA, were readily incorporated by *S*. *aureus*, as shown by increased label-specific fluorescence (Fig 1B). While the entire cell surface appeared to incorporate LAHA, there was a marked zone of higher-intensity fluorescence at the equatorial cleavage plane of the cells (Fig 1C).

Because log-phase and stationary-phase bacteria show distinct patterns of gene expression and protein synthesis, we tested if cells in either growth phase were capable of incorporating LAHA into surface-exposed proteins. We incubated stationary or log-phase cultures for one hour with LAHA or DMSO control, washed out excess label, and detected LAHA incorporation using DM543. We found that *S*. *aureus* incorporated LAHA during stationary phase as well during log phase, although to a lesser extent (Fig 1D).

**Fig 2:**
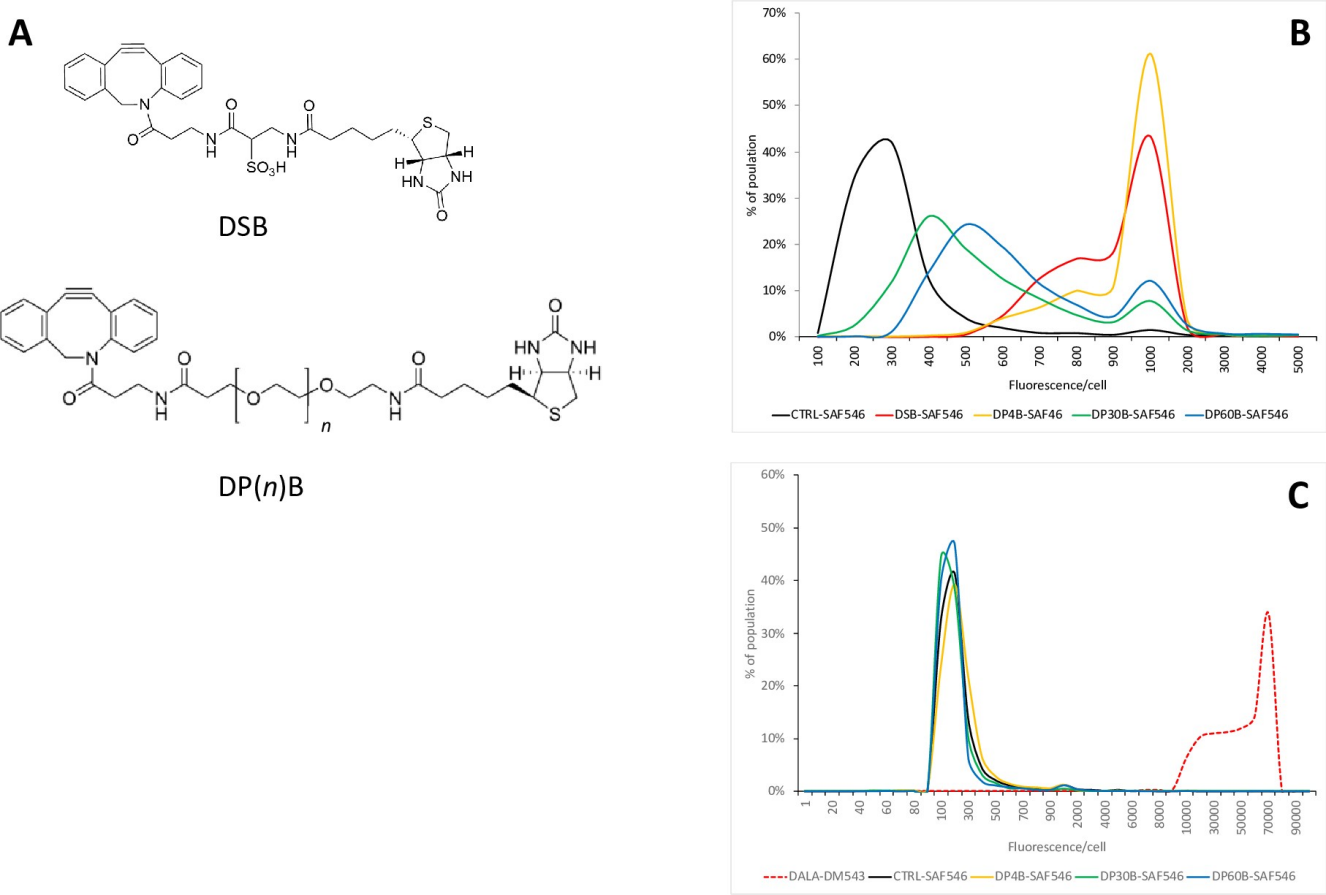
LAHA, but not DALA, is available for streptavidin binding on metabolically labeled cells. (A) Structure of a water-soluble DBCO-sulfo-biotin (DSB), an octyne-biotin with a short, rigid linker, and structure of DBCO-PEGn-Biotin; the PEG linkers tested in this study ranged from 4–60 PEG repeats. (B) Short linker-length octyne-biotins support streptavidin binding to LAHA labeled cells. All cells were labeled for 1 hour with 5 mM LAHA, without (CTRL) or with the indicated octyne-biotin for 15 minutes, followed by streptavidin-alexafluor 546 (SAF546); DSB = DBCO-sulfo-biotin; DP4B = DBCO-PEG4-biotin; DP30B = DBCO-PEG30-biotin; DP60B = DBCO-PEG60-biotin. (C) DALA is accessible for click reaction, but not streptavidin binding. All cells were labeled for 1 hour with 5 mM DALA, without (CTRL) or with the indicated octyne-biotin for 15 minutes, followed by SAF546; a parallel DALA-labeled sample was incubated with the octyne-fluorophore DM543 to demonstrate the incorporation of DALA. Octyne-biotin designations are the same as those shown in panel A. n = 3 for each experiment; a representative example is shown.

Our label-screening experiments established that both LAHA and DALA are incorporated and solution exposed on *S*. *aureus*. To determine if the azido groups of these molecules were accessible for use in an enrichment method, we tested a panel of DBCO-biotin click reagents with structurally diverse linkers for streptavidin binding. DBCO-sulfo-biotin (DSB, Fig 2A) has a short, rigid linker separating the reactive octyne and the biotin moieties. DBCO-biotins with flexible polyethyleneglycol (PEG) linkers of length 4, 30, 60, or 100 PEG repeats (DP4B, DP30B, DP60B, and DP100B, respectively, Fig 2B) were also tested. Following metabolic labeling in log-phase and washout of unincorporated label, cells were subjected to SPAAC with the various DBCO-biotins. Unreacted DBCO-biotin was washed out, and cells were incubated with streptavidin-AlexaFluor-546 (SAF546). Biotin accessibility was determined by fluorescence microscopy.

We found that LAHA metabolically labelled cells could be specifically stained with streptavidin fluorophore of all linker lengths, but that there was a weak inverse correlation between fluorescence intensity and the linker length of the octyne-biotin (Fig 2C). However, no fluorescence above background was detectable in DALA-labeled cells using a streptavidin fluorophore (Fig 2D). This is stark difference between direct and indirect fluorescence detection of DALA incorporation is probably because the dense structure of the cell wall is permeable to the comparatively small DM543, but not the sterically bulky streptavidin protein.

To establish the feasibility of metabolic labeling in a patient sample, we examined LAHA incorporation in *S*. *aureus* spiked into whole blood. After addition of bacteria, host cells were lysed by a detergent-enzyme cocktail that specifically permeabilizes eukaryotic cells, incubated with pronase and DNase I to degrade host proteins and nucleic acids. Cells were treated with LAHA or vehicle control in the lysed blood for 1 hour and pelleted by centrifugation. The supernatant containing unincorporated label was removed, and the pellet was washed. LAHA incorporation was probed by direct fluorescence following SPAAC of DM543.

**Fig 3:**
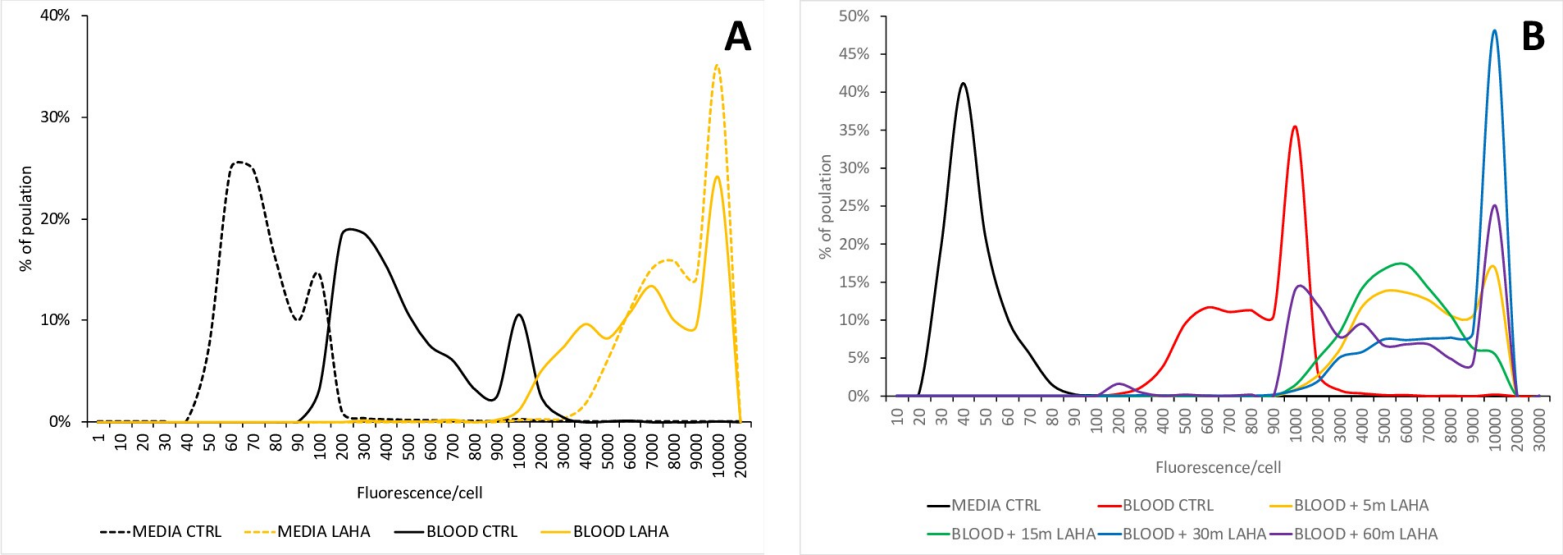
LAHA can label cells in whole blood. **(**A) Fluorescent labeling of control or LAHA-labeled *S*. *aureus* in media versus blood. Cells were grown for 1 hour in minimal medium (MEDIA, broken lines) or blood (BLOOD, solid lines) with LAHA (yellow lines) or DMSO control (CTRL, black lines); after washout, cells were exposed to DM543 for 15 minutes, and imaged by fluorescence microscopy. (B) LAHA is rapidly incorporated by cells growing in blood; cells growing in blood were exposed to 5 mM LAHA for 5 minutes (yellow), 15 minutes (green), 30 minutes (blue), or 60 minutes (purple), followed by exposure to DM543 for 15 minutes; cells grown in blood without LAHA followed by 15 minutes exposure to DM543 served as a fluorescence control (BLOOD CTRL, red line); cells grown in medium serve as a control for background fluorescence (MEDIA CTRL, black line). n = 2 for each experiment; a representative example is shown.

We found that *S*. *aureus* was efficiently labeled by LAHA in whole blood, to an extent comparable to that observed in minimal medium. Unlabeled cells incubated in whole blood showed moderately higher background fluorescence than unlabeled cells grown in minimal medium, possibly due to the adherence of auto-fluorescent molecules from whole blood to *S*. *aureus* cell wall (Fig 3A).

Because speed is a critical factor in any sepsis diagnostic test, we next determined the minimum duration of LAHA exposure necessary for specific labeling of cells in whole blood. As in the experiment described above, *S*. *aureus* was inoculated into whole blood, and the inoculum was treated with a detergent-enzyme cocktail. A negative control aliquot was removed from the sample, collected by centrifugation, and the pellet was washed to remove unincorporated label. The extent of label incorporation was determined by SPAAC-mediated addition of DM543. We found that a treatment duration of as little as 5 minutes was sufficient to label the entire population of cells. Interestingly, we observed an increase in the number of cells with low fluorescence intensity after 1 hour of metabolic labeling, which may reflect dilution of the label due to cell division during the incubation period (Fig 3B).

**Fig 4:**
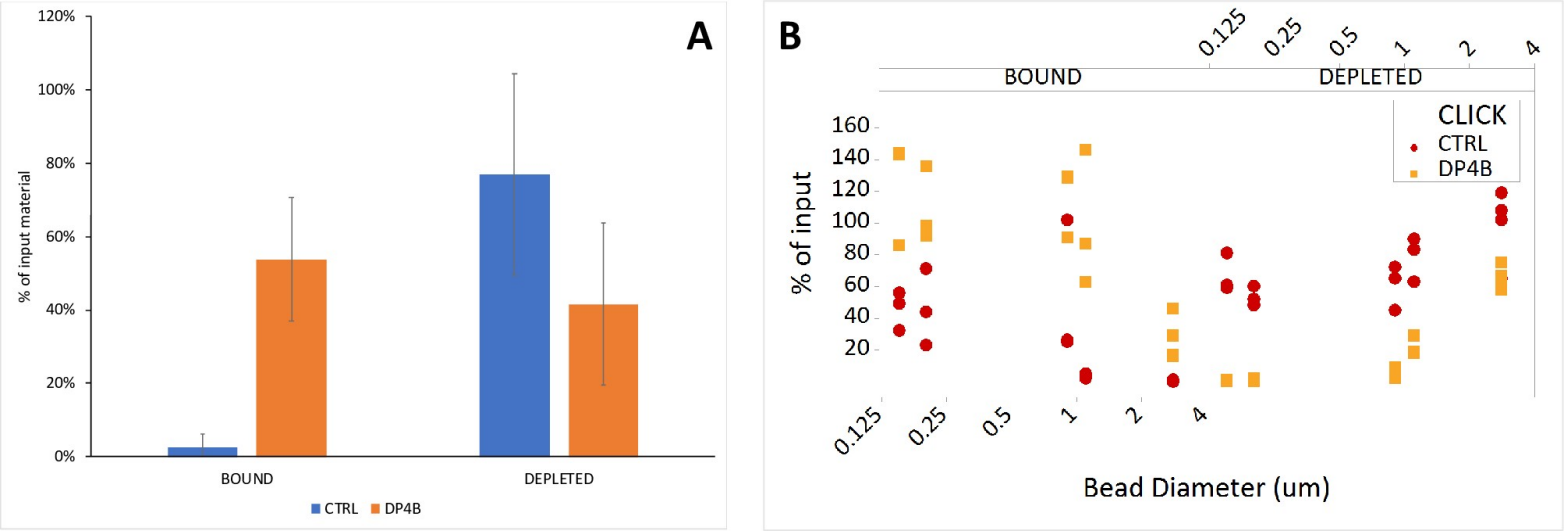
Metabolic labeling with LAHA can be used to deplete viable cells from complex starting samples. (A) *S*. *aureus* in whole blood (10^2^−10^4^ CFU from a titered stock) was incubated with LAHA for 15 minutes, followed by exposure to DBCO-PEG4-Biotin (DP4B) or DMSO (CTRL). The labeled cells were incubated with 2.8 μm diameter Streptavidin M280 beads for 30 minutes with continuous agitation, and collected on a magnet; bead-bound and depleted fractions were tested for the presence of viable cells by plating compared to an input control sample; click-biotinylation allows for significant depletion of labeled cells relative to control (p < 0.01, t-test; n = 10). (B) Streptavidin bead diameter is a critical determinant of enrichment/depletion efficiency. Cells, grown as in panel A, were incubated with magnetic beads of varying diameters (see Table 1 for comparative physical characteristics of all bead types), and the number of colony-forming units as a percent of the input was calculated by growth on solid media. DP4B (gold squares) = LAHA-labeled and DP4B biotinylated cells; CTRL (red circles) = LAHA-labeled control cells without biotinylation.

Next, we sought to use metabolic labeling and biorthogonal cycloaddition to specifically enrich bacteria from whole blood with minimal additional processing. Using a DP4B, we biotinylated cells that were metabolically labeled in a whole-blood inoculum treated with detergent-enzyme cocktail as described above. After washout of unincorporated reagents, we bound biotinylated cells to streptavidin M280 magnetic beads, and collected using a neodymium magnet. Total bacteria used as input, recovered after binding from the supernatant, and attached to the beads were enumerated based on the colony forming assay (see Materials and methods). Enrichment or capture efficiency was determined by dividing colony formation units determined for the bead-bound fraction colony formation units determined for the input. An enrichment efficiency of 55% (range 37–77%) was seen in labeled and biotinylated cells, compared to 0% enrichment for cells that were labeled but not biotinylated (Fig 4A).

Because bead-based enrichment methods rely on the stochastic collisions between biotinylated cells and the streptavidin capture support, we reasoned that capture efficiency could be improved using an alternative bead geometry. Table 1 summarizes the binding capacity and surface area-per-unit mass of a variety of commercially available streptavidin magnetic beads.

**Table 1:** Physical properties of a selection of commercially available streptavidin magnetic beads.

Bead	M-280	MyOne T1	HiSur-L	HiSur-S	SuperMag
**Binding capacity, nmol/mg**	0.65	1.3	7	7	1.7
**Diameter,** μ**m**	2.8	1.0	1.0	0.15	0.2
**Magnetic content, %w/v**	12%	26%	60%	70%	80%
**Beads/mg**	6.3x10^7^	1x10^9^	1.2x10^9^	4x10^12^	1.3x10^11^
**Surface area, cm**^**2**^**/mg**	15.51	31.40	>37.68	>2840	169
**Surface geometry**	Smooth	Smooth	Rough	Rough	Smooth

Surface area per unit mass is inversely proportional to bead diameter and is further determined by surface geometry (rough or smooth).

We subsequently characterized the capture efficiency of each bead type for biotinylated and control cells that were metabolically labeled in a whole-blood inoculum treated with detergent-enzyme cocktail as described above. We found that for equivalent masses of streptavidin magnetic resin, enrichment efficiency increased with decreasing bead diameter and increased surface area (Fig 4B). However, this was offset by an increase in non-specific binding of cells that were not biotinylated by small bead diameter/high surface area resins. Based on our testing, the 1 μm diameter MyOne T1 streptavidin beads represent an optimal combination of low background binding and high capture efficiency. Overall, biotinylation of LAHA-labeled cells substantially improved enrichment relative to controls, as determined by the comparing the number of colony-forming units in the bound fraction relative to the input fraction for biotinylated and non-biotinylated cells. The extent of supernatant depletion relative to input was comparable to the extent of enrichment seen in the bound fraction. These data are summarized in Table 2.

**Table 2:** Specific enrichment/depletion of LAHA-labeled, biotinylated *S*. *aureus* using streptavidin magnetic beads.

Bead Type	cm^2^/mg	n	Bound (% of Input ± SD)			Depleted (% of Input ± SD)		
			Ctrl	DP4B	*p-*value	Ctrl	DP4B	*p-*value
**M280**	15.5	4	0 ± 1%	30 ± 12%	0.06	98% ± 24%	66% ± 7%	0.023
**MyOne**	31.4	3	6 ± 5%	92± 27%	<0.001	79% ± 14%	22% ± 6%	0.001
**HiSur L**	37.7	3	51± 44%	112 ± 19%	<0.001	60% ± 14%	5% ± 4%	<0.001
**SuperMag**	169	3	45 ± 14%	105 ± 10%	<0.001	53% ± 6%	1% ± 1%	0.002
**HiSur S**	2840	3	46 ± 12%	116± 29%	<0.001	67% ± 12%	1% ± 1%	0.001

Enrichment and depletion efficiency of control and biotinylated LAHA-labeled *S*. *aureus*. Bound % is calculated as (CFU bound/CFU input)*100%, and depleted % is calculated as (CFU depleted/CFU input)*100%. Differences in the means were calculated by one-way ANOVA, and *p*-values were determined using Fisher’s PLSD.

## Conclusions

In this study, we describe a method for labeling and enriching viable *S*. *aureus* from complex biological samples with limited processing. By screening a panel of commercially available metabolic labeling reagents, we identified two, L-azido-homoalanine (LAHA) and Azido-D-alanine (DALA), that could rapidly label cells. Furthermore, we have shown that newly-synthesized, metabolically-labeled proteins are presented on the cell surface and accessible for binding by streptavidin after biotinylation. Moreover, we establish protein metabolic labeling and biotinylation as an effective method for isolation of *S*. *aureus* and identified characteristics of linker structure and resin properties that determine binding efficiency. The entire workflow can be completed within 1 hour from crude sample to captured bacteria, which are viable and can be used as an input for downstream assays.

The applicability of biorthogonal methods to bacterial enrichment has been shown previously [28]. However, the existing methods are less attractive for rapid diagnostic applications because of the long exposure duration required for incorporation of Kdo-Azide. Furthermore, Kdo-Azide is biologically restricted to use with gram-negative organisms. By contrast, the method described in this study permits labeling within minutes rather than hours, and while tested here with *S*. *aureus*, could in principle be extended to additional gram-positive and possibly gram-negative organisms.

In developing our workflow, we found that the linker length separating the reactive octyne from the biotin prey moiety is a critical factor determining streptavidin binding to labeled cells. Octyne-biotins with shorter linker lengths may have improved diffusion characteristics, allowing reactions to occur in areas of the cell wall or outer membrane that are otherwise inaccessible to octyne-biotins with longer linkers. Furthermore, the combinatorial synthesis or use of octyne-biotins with diverse linker properties would allow for the addition of multiple functionalities to LAHA-labeled cells. For example, chemical- or photo-cleavable linkers would allow for the release of labeled cells from the capture resin, directly to a molecular identification assay.

We also found that the diameter and surface properties of the streptavidin beads used for enrichment was a major factor in determining overall enrichment efficiency. Reducing bead diameter dramatically improved capture efficiency for the same mass of resin. One intriguing avenue for future exploration would be the use of cyclooctyne-derivatized magnetic nanoparticles [29], which would allow for the direct coupling of labeled cells and capture support in a single step. This would further simplify the described method by eliminating additional handling steps related to cleanup of unincorporated click reagents.

Rapid identification and genotypic antibiotic susceptibility testing of *S*. *aureus* is a critical unmet clinical need [8, 16, 18]. Because of technical limitations imposed by pathogen abundance and sample composition, current methods for identification of bloodstream infections of *S*. *aureus* require extended culture steps that are incompatible with the critical nature of sepsis care. As a consequence, empiric treatments are offered using a suite of diminishingly effective antibiotics of last resort [3, 6, 7]. This has profound long-term consequences for both patient recovery and the spread of antibiotic resistance. Shifting to a more targeted course of antibiotics during the early stages of sepsis care could provide real benefits for infection control, antibiotic stewardship, as well as dramatic reductions in the overall cost of care [8, 15]. If coupled with a rapid genotyping test, the method described here could provide strain-level information about *S*. *aureus* within the duration of a single hospital shift, a substantial improvement over the current standard of care.

Current methods of post-culture genotyping rely on upwards of 10^6^ CFU/mL of pathogens to provide strain identification and antibiotic susceptibility information [17, 30, 31]. By contrast, it is estimated that septic individuals can have a pathogen burden below 1 CFU/mL with a fraction being between 10s-100s of CFU/mL [32]. The method described here has been shown to capture viable *S*. *aureus* in the range of 50−10^4^ CFU/mL, placing it closer to a clinically-relevant detection range, especially compared to other existing methods. If coupled with a rapid genotyping test, the method described here could provide strain-level information about *S*. *aureus* within the duration of a single hospital shift, a substantial improvement over the current standard of care. Further work will be needed to test lower ranges of bacterial loads of various clinical isolates, higher volume of blood, and using more sensitive output methods other than plating for quantification of the enrichment efficiency.

In summary, the work described here establishes a method for enriching live *S*. *aureus* based on incorporation of amino acid analogs into newly-synthesize proteins. It has potential for use in downstream diagnostic applications that require viable bacteria, such as pathogen identification or (genotypic) antibiotic susceptibility testing, without an intervening culture step.
